# Interface Engineering in All-Oxide Photovoltaic Devices Based on Photoferroelectric BiFe_0.9_Co_0.1_O_3_ Thin Films

**DOI:** 10.1021/acsaelm.4c01533

**Published:** 2024-11-13

**Authors:** Pamela Machado, Pol Salles, Alexander Frebel, Gabriele De Luca, Eloi Ros, Christian Hagendorf, Ignasi Fina, Joaquim Puigdollers, Mariona Coll

**Affiliations:** † Institut de Ciència de Materials de Barcelona, ICMAB-CSIC, Campus UAB, Bellaterra 08193, Spain; ‡ Department of Materials and Geo Sciences, Surface Science Division, Technische Universität Darmstadt, Jovanka-Bontschits-Straße 2, D-64287 Darmstadt, Germany; § Catalan Institute of Nanoscience and Nanotechnology (ICN2), Campus UAB, Bellaterra 08193, Barcelona, Spain; ∥ Departament d’Enginyeria Electrònica, Universitat Politècnica de Catalunya, Jordi Girona 1–3, Barcelona 08034, Spain; ⊥ Fraunhofer Center for Silicon-Photovoltaics, CSP, Otto-Eissfeldt-Strasse 12, 06120 Halle (Saale), Germany

**Keywords:** oxides, photoferroelectrics, thin films, interface, BiFeO_3_

## Abstract

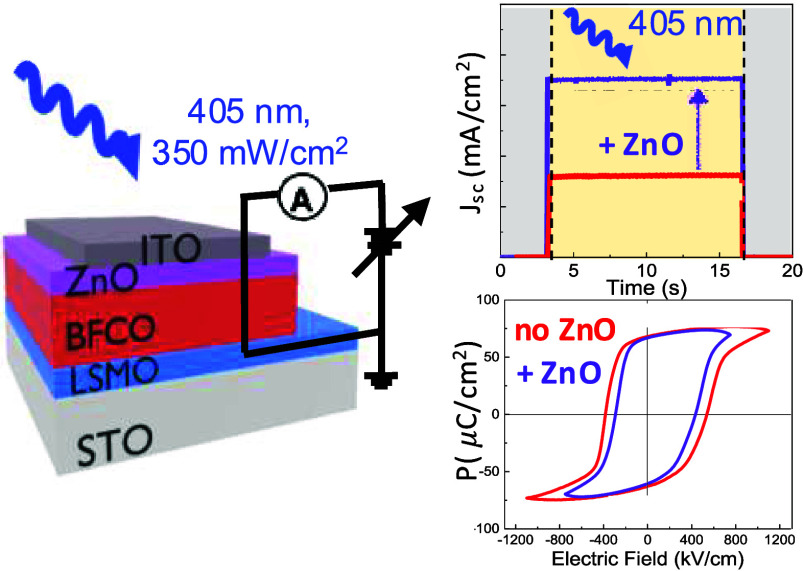

Photoferroelectric BiFeO_3_ (BFO) has attracted renewed interest to be integrated into thin film photovoltaic (PV) devices as a stable, lead-free, and versatile photoabsorber with simplified architecture. While significant efforts have been dedicated toward the exploration of strategies to tailor the properties of this photoabsorber to improve the device performance, efficiencies still remain low. The modification of the BFO interface by the incorporation of transport-selective layers can offer fresh opportunities to modify the properties of the device. Identifying an optical and electrically suitable selective layer while ensuring easy device processing and controlled film properties is challenging. In this work, we determine the influence of incorporating a ZnO layer on the ferroelectric and photoresponse behavior of an epitaxial BiFe_0.9_Co_0.1_O_3_ (BFCO)-based heterostructure. The device is completed with Sn-doped In_2_O_3_ (ITO) and La_0.7_Sr_0.3_MnO_3_ (LSMO) electrodes. This all-oxide system is stable under ambient conditions and displays robust ferroelectricity. The coupled ferroelectricity–photoresponse measurements demonstrate that the short circuit current can be modulated by ferroelectric polarization in up to 68% under blue monochromatic light. Also, the responsivity of the system with the ZnO-modified interface is larger than that of the system with no ZnO. Complementary band energy alignment studies reveal that the observed increase in the short circuit current density of the device with ZnO is attributed to lower Fermi level energy at the ZnO/BFCO interface compared to the ITO/BFCO interface, which reduces charge recombination. Therefore, this study provides useful insights into the role of the ZnO interface layer in stable BFO-based devices to further explore their viability for potential optoelectronic applications.

## Introduction

All-oxide photovoltaics (PV), envisaging a solar cell entirely based on thin film oxides, emerges as an attractive approach to complement the current PV technologies. In addition to versatility in composition, structure and physical properties, it offers the possibility to use stable and low-toxic elements prepared by cost-effective routes.^1−3^ Photoferroelectric perovskite oxide-based solar cells have sparked a great deal of interest because their unique bulk PV effect could open the possibility of surpassing the fundamental efficiency limits of traditional semiconductors using simplified device architectures.^4,5^ Additionally, the ferroelectric polarization switching of these materials could offer an extra mechanism to modulate and potentially reverse charge transport properties.^6−11^

BiFeO_3_ (BFO) has been largely investigated as a lead-free light absorber with a relatively narrow band gap (∼2.7 eV) and high ferroelectric polarization at room temperature,^12−15^ but it only allows to harvest 8–20% of the solar spectrum. Different strategies based on the engineering of the BFO layer have been evaluated to boost the device performance;^16−23^ however, PV efficiencies still remain low.^14^ In traditional and emergent PV technologies, the use of optically, chemically, and electrically compatible charge transport layers and electrodes helps promote the traveling and collection of photogenerated carriers and improves the device performance.^24−26^ Recently, the implementation of these suitably matched interfacial layers has started to be investigated in photoferroelectric polycrystalline oxide perovskite systems. For example, ZnO, a typical electron transport layer used in hybrid perovskite and chalcogenide solar cells, has been evaluated owing to its high carrier mobility, matching band gap, and easy processability.^27−29^ In less extend, other interfacial materials such as WS_2_^30^ and reduced-graphene oxide,^31^ have also been investigated, although the material dissimilarity can add complexity in BFO device integration and processing.^32^ Nonetheless, the study of the influence of interface engineering to modulate the photoresponse in epitaxial BFO films has remained elusive, although epitaxy has been key to controlling its multiferroic and optical properties through strain engineering.^33^

**Figure 1:**
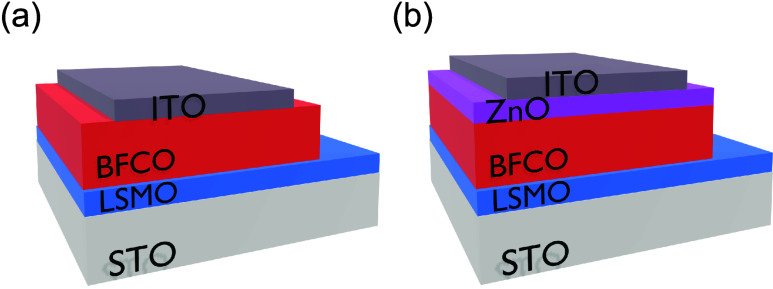
Sketch of the all-oxide architectures investigated: (a) ITO/BFCO/LSMO//STO and (b) ITO/ZnO/BFCO/LSMO//STO.

Here, we report the preparation of epitaxial BFO-based devices and the influence of the ZnO interface layer on the photoferroelectric response. In particular, a BiFe_0.9_Co_0.1_O_3_ (BFCO) photoactive layer with an engineered optical band gap of 2.6 eV and high remnant ferroelectric polarization at room temperature (60 μC/cm^2^) has been studied.^17,34^ The designed oxide heterostructure consists of top electrode/ZnO/BFCO/bottom electrode, where Sn-doped In_2_O_3_ (ITO) and La_0.7_Sr_0.3_MnO_3_ (LSMO) have been selected for top and bottom electrodes, respectively (see Figure 1). The stack is grown on SrTiO_3_ (STO) single-crystal substrates to promote the epitaxial growth of LSMO and BFCO, prioritizing the use of cost-effective deposition techniques. The analogous system with no ZnO is also fabricated for comparison. X-ray diffraction analysis confirmed the epitaxial growth of LSMO and BFCO layers, whereas atomic force microscopy revealed the formation of films with homogeneous, granular, and smooth surface morphology after the deposition of four additive layers. Macroscopic ferroelectric polarization loops show minimal differences after interface engineering, whereas current density–voltage (*J*–*V*) characteristics disclosed enhanced performance in the heterostructure with ZnO. Complementary X-ray photoelectron spectroscopy analysis provides additional information on the energy band alignment of these oxide heterostructures, identifying a slight shift of the Fermi energy for BFCO/ZnO compared to BFCO/ITO.

## Experimental Section

### Thin Film Fabrication

#### LSMO Films

Stoichiometric amounts of lanthanum, strontium, and manganese acetates were dissolved in a solvent blend of acetic acid and water (5:1) to obtain 0.1 M LSMO solution. The precursor solution was subsequently spin-coated on 5 mm × 5 mm (00*l*)-oriented STO substrates cleaned with ethanol, followed by N_2_ blow dry. The deposition conditions were 6000 rpm for 30 s at ambient conditions and a maximum relative humidity of 30–40%. The sample was subsequently exposed to crystallization treatment at 900 °C for 15 min in an O_2_ atmosphere.^35^ These procedures resulted in 10 nm LSMO thin films.

BFCO films were prepared from a precursor solution of stoichiometric amounts of metal nitrates as established elsewhere.^34^ The viscosity of the solution was 0.003 Pa·s, measured at 22 °C at a fixed speed of 2880 s^–1^ for 30 s using a Haake RheoStress RS600 rheometer from Thermo Electron Corporation. The thickness of the BFCO film was adjusted by modifying the molar concentration of the precursor solution and/or performing multideposition.

ZnO films were prepared by atomic layer deposition using a commercial reactor from a Savannah-Cambridge Nanotech 100. Diethylzinc and Milli-Q water were alternately pulsed/purged for 0.15/20 s and 0.015/20 s, respectively, under a 20 sccm N_2_ carrier gas. The reactor temperature was set at 160 °C, and the growth rate was 1.1 ± 0.2 Å/cycle.

ITO films were deposited by radio frequency magnetron sputtering using an In_2_O_3_:SnO_2_ 90:10 wt % target of 1.5 in. in diameter in an on-axis configuration at a working distance of 15 cm. The deposition was carried out at room temperature with an estimated growth rate of 0.6 nm/min and a sputtering power density of 2.2 W·cm^2^. A working pressure of ∼2 mTorr was stabilized with a constant Ar flow of 10 sccm, and a butterfly valve operated in static mode. For the device fabrication, a grid was used as a shadow mask to obtain an array of contacts of 40 μm × 40 μm and 130 μm × 130 μm for ferroelectric and photoresponse measurements, respectively.

Two oxide heterostructures have been prepared to investigate the effect of ZnO on the ferroelectric and photoresponse behavior: ITO(70 nm)/ZnO(20 nm)/BFCO(100 nm)/LSMO(10 nm)//STO and ITO(70 nm)/BFCO(100 nm)/LSMO(10 nm)//STO. The thickness of the different components was verified by cross-sectional images from transmission electron microscopy (TEM), X-ray reflectometry, and profilometry (Figure S1).

Specifics of the sample configuration designed for energy band alignment studies are detailed in the XPS Characterization section.

### Characterization of Films and Heterostructures

The crystallinity and phase purity of the oxide heterostructures were analyzed by means of X-ray diffraction (XRD). XRD θ–2θ scan measurements were performed in the range of 20–80° using a Bruker-AXS A25 D8 Discover equipped with a Cu anode (Cu Kα λ = 1.5418 Å) and θ–χ scans using a Bruker D8 Advance general area detector diffraction system (GADDS) equipped with a 2D Vantec 500 detector.

Surface morphology and root-mean-square (rms) roughness were studied from the topography images acquired with an atomic force microscope (AFM, Keysight 5100 instrument) in dynamic mode and analyzed by MountainsMap Premium 9 software from Digital Surf.^36^

#### Optical Properties

The ultraviolet–visible (UV–vis) optical transmittance and absorbance spectra were studied on a UV–vis/NIR spectrophotometer from Jasco V780 in the wavelength range of 200–800 nm, with a scan speed of 400 nm/min and in continuous scan mode at normal incidence. All measurements were performed on samples prepared on one-side polished STO substrates. The contribution of the substrate was not subtracted. Transmittance map simulations of the ITO/ZnO/BFCO//STO system were obtained by the transfer matrix method at 400 nm irradiance conditions and considering the refractive index and extinction coefficient of ITO, ZnO, and BFCO films (see Figure S2).

Variable-angle spectroscopic ellipsometry (SE) measurements were performed for optical band gap evaluation using a SOPRALAB GES5E ellipsometer equipped with a Xe lamp as the light source and a charge-coupled device detector. Ellipsometric spectra were collected at room temperature, with the analyzer angle fixed at 45° and under different angles of incidence (65, 68, 70, 72, and 75°) in the energy range of 1.2–4.5 eV. Acquired data were analyzed using WinElli II software, modeled with the structure void/film/substrate and using Tauc-Lorentz and Cauchy optical models to describe the optical properties for the films as described elsewhere.^17,37,38^ For optical band gap determination of ZnO and BFCO, ZnO(25 nm)//SiO_2_/Si and BFCO(25 nm)//STO were prepared, respectively.

#### Photoresponse

The current density versus voltage (*J*–*V*) electrical characteristics of the complete devices were measured using a Keithley 2517B electrometer, applying a sweeping voltage of ±0.4 V at the top ITO electrode and grounding the LSMO bottom contact. This sweeping voltage was lower than the coercive electric field of BFCO, ensuring no switching during the *J*–*V* measurement of the curve. For the time-dependent short circuit current density (*J*_sc_–time) measurements, an aixACCT TF Analyzer 2000 system was used. Both *J*–*V* and *J*_sc_–time photoresponse measurements were carried out illuminating from the top of 130 μm × 130 μm ITO electrodes (70 nm) using a blue monochromatic laser with a wavelength of 405 nm and a power irradiance of 350 mW/cm^2^. The laser irradiated the sample at an angle of 45°. The spot diameter was approximately 400 μm, safely illuminating homogeneously two adjacent electrodes, as described in ref (39).

The *J*–*V* characteristics of the ITO/ZnO/BFCO/LSMO//STO with different ZnO thicknesses (4–130 nm) were acquired under illumination to confirm optimal ZnO film thickness (20 nm; see Figure S3). The photoresponse measurements were carried out in as-grown and prepolarized systems, where upward and downward polarization states were defined when negative and positive voltages were applied to the top of the ITO electrode, respectively. For prepolarized systems, *J*–*V* curves were collected under illumination with a time delay of ∼10 s after applying a triangular polarization voltage (*V*_poling_) of up to ±6 V. In addition, the dependence of *J*_sc_ with *V*_poling_ was recorded by applying a *V*_poling_ of up to ±6.5 V to the top of ITO and collecting the current density under illumination at zero voltage with a time delay of 0.1 s for each voltage applied. The incident photon to current efficiency (IPCE) was calculated to define how efficiently the devices convert the incident light into electricity at a given wavelength,^40^ following eq 1
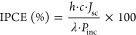
1where λ and *P*_inc_ correspond to the wavelength and power irradiance of the incident monochromatic light, respectively, *h* is Planck’s constant, and *c* is the velocity of light.

The results shown here are the averages of the best measurements from more than 150 devices from identical samples prepared in three different batches.

#### Ferroelectric Properties

Ferroelectric polarization versus electric field (*P*–*E*) and current density versus electric field (*J*–*E*) loops were measured at room temperature in as-prepared systems using the TF Analyzer 2000 platform, sweeping electric fields of 750 kV/cm (ZnO/BFCO system) and 1100 kV/cm (BFCO system) to the top of 40 μm × 40 μm ITO electrodes (70 nm) at a constant rate with a frequency of 2 kHz and grounding the bottom electrode LSMO using the virtual ground method (see the TF Analyzer 2000 user manual). The dynamic leakage current compensation was used to minimize leakage current effects.^41^ Polarization was obtained by displacive current integration over time and normalized to the electrode area.

#### XPS and Band Alignment Determination

The study of core level binding energies and valence band maxima (VBM) of the pristine oxide surfaces and interfaces was carried out by X-ray photoelectron spectroscopy (XPS)^42^ using a PHOIBOS 150 hemispherical electron analyzer (SPECS GmbH) at a base pressure of 5–8.5 × 10^–10^ mbar with a monochromatic Al Kα radiation (1486.74 eV) source operated at 300 W. The energy resolution as measured by the full width at half-maximum (fwhm) of the Ag 3d_5/2_ peak for a sputtered silver foil was 0.62 eV. Data processing was performed with CasaXPS software. The characteristics of the pristine surface of ITO, ZnO, LSMO, and BFCO were studied from ITO(50 nm)//SiO_2_/Si, ZnO(25 nm)//SiO_2_/Si, BFCO(100 nm)/(10 nm)LSMO//STO, and LSMO(10 nm)//STO, respectively. The characteristics of the different interfaces existing in the heterostructures were studied from ITO(3 nm)/BFCO(100 nm)/LSMO (10 nm)//STO, ZnO(5 nm)/BFCO(25 nm)/LSMO (10 nm)//STO, BFCO(5 nm)/LSMO(10 nm)//STO, and ITO(3 nm)/ZnO(25 nm)/SiO_2_/Si.

The binding energies were measured with respect to the Fermi energy of the analyzer, which was calibrated by measuring an atomically clean Au/glass sample. The binding energy positions and shifts were determined by the maxima of the fitted spectra. Samples were momentarily exposed to air when removed from the synthesis equipment and brought to the XPS sealed in vacuum. The core level binding energies for ITO (In 3d, Sn 3d), ZnO (Zn 2p), BFCO (Bi 4f), and LSMO (Sr 3d) and the respective valence band maxima (VBM) were extracted.

The valence band offset (Δ*E*_VBM_) values at ITO/BFCO, ZnO/BFCO, and BFCO/LSMO interfaces were obtained by measuring the energy differences associated with the relative position to the VBM and core levels of the different materials. The study was completed by extracting the conduction band minima (CBM) and conduction band offset (Δ*E*_CBM_) using the energy gap (*E*_g_). The *E*_g_ of BFCO and ZnO were determined experimentally by spectroscopic ellipsometry, whereas the value for ITO was extracted from the literature.^43,44^ Details on the data analysis can be found in the Supporting Information (eqs S1–S3, Table S1, and Figures S9 and S10).

## Results

**Figure 2:**
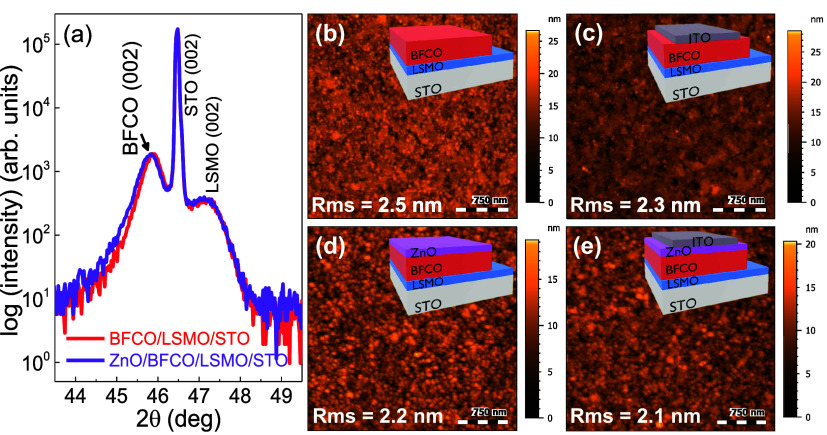
(a) XRD θ–2θ scan of ITO/BFCO/LSMO//STO (red) and ITO/ZnO/BFCO/LSMO//STO (purple). AFM topography images of (b) BFCO on LSMO//STO, (c) ITO on BFCO/LSMO//STO, (d) ZnO on BFCO/LSMO//STO, and (e) ITO on ZnO/BFCO/LSMO//STO with the corresponding architecture as the inset.

The film crystallinity and surface morphology have been investigated for the two all-oxide systems: ITO(70 nm)/BFCO(100 nm)/LSMO(10 nm)//STO and ITO(70 nm)/ZnO(20 nm)/BFCO(100 nm)/LSMO(10 nm)//STO (Figure 2). The XRD θ–2θ studies on the complete devices show three Bragg reflections corresponding to (002) STO (2θ = 46.5°), (002) LSMO (2θ = 47.2°), and (002) BFCO (2θ = 45.9°), suggesting *c*-axis-oriented growth for BFCO and LSMO (Figure 2a). Note that the experimental out-of-plane lattice parameter for 100 nm BFCO is ∼3.95 Å, indicating a fully relaxed film.^17^ Additional 2D XRD θ–χ scans confirmed the epitaxial growth of BFCO and LSMO and the polycrystalline nature of ZnO (see Figure S4). AFM topography images collected on the surfaces of BFCO/LSMO//STO, ITO/BFCO/LSMO//STO, ZnO/BFCO/LSMO//STO and ITO/ZnO/BFCO/LSMO//STO heterostructures show smooth, granular, and homogeneous surfaces with an average rms roughness of 2.3 ± 0.2 nm (Figure 2b–e).

**Figure 3:**
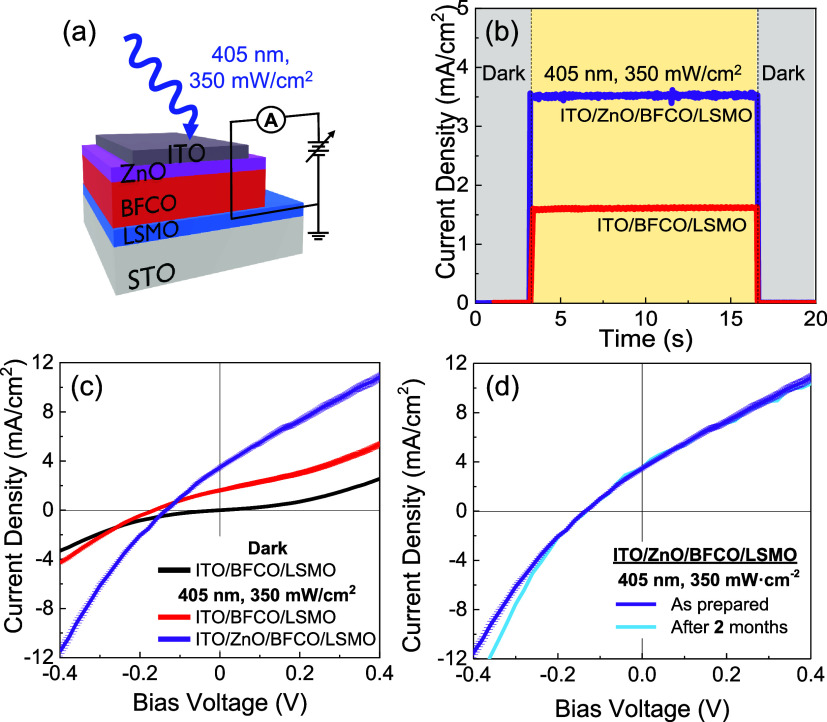
(a) Sketch of the electrical configuration used to measure the photoresponse of BFCO-based systems. (b) Time-dependent *J*_sc_ and (c) *J*–*V* characteristic curves of ITO/BFCO/LSMO//STO (red) and ITO/ZnO/BFCO/LSMO//STO (purple). (d) Comparative *J*–*V* curves of ITO/ZnO/BFCO/LSMO//STO measured in a time interval of 2 months to test the operational stability of the sample.

The influence of the ZnO layer on the photoresponse of the BFCO-based heterostructure has been investigated by illuminating the samples from the top ITO electrode with a monochromatic laser of 405 nm and an irradiance of 350 mW/cm^2^; see the device configuration sketched in Figure 3a, and compared to the same heterostructure with no ZnO. The time-dependent *J*_sc_ is measured in dark and under laser illumination for ∼10 s, exhibiting a stable and fast photoresponse that significantly increases upon the integration of the ZnO interface (Figure 3b). Figure 3c shows the current density–voltage (*J*–*V*) characteristics in dark and under illumination, revealing a diode-like shape as well as a clear photovoltaic response upon illumination, consistent with the design of an asymmetric and rectifying junction.^13,16^ The resulting *J*_sc_, open circuit voltage (*V*_oc_), and IPCE (eq 1) are presented in Table 1. The magnitude of *J*_sc_ in ITO/ZnO/BFCO (3.5 mA/cm^2^) is ∼2.2 times larger than that in ITO/BFCO (1.6 mA/cm^2^), which leads to an IPCE of up to 4.4% (405 nm). In order to rule out the possibility that *J*_sc_ improvements arose from the photogeneration in ZnO as is reported in polycrystalline BFO systems,^27,45^*J*–*V* curves have been collected in the ITO(70 nm)/ZnO(20 nm)/LSMO(10 nm)//STO system under the same measuring conditions. It turns out that no photogeneration occurs in ZnO, as shown in Figure S5. On the other hand, the addition of the ZnO interface layer to the BFCO device does not significantly modify the *V*_oc_, whose value modestly shifts from −0.19 V in the ITO/BFCO system to −0.14 ± 0.01 V in ITO/ZnO/BFCO.

**Table 1:** Comparison of *J*_sc_, *V*_oc_, IPCE, and R Parameters from *J*–*V* Characteristics of ITO/BFCO/LSMO//STO and ITO/ZnO/BFCO/LSMO//STO

system	*J*_sc_ ± 0.1 (mA/cm^2^)	*V*_oc_ ± 0.01 (V)	IPCE (%)	*R* (A/W)
ITO/BFCO/LSMO//STO	1.6	–0.19	2.0	4.6 × 10^–3^
ITO/ZnO/BFCO/LSMO//STO	3.5	–0.14	4.4	0.01

It is of interest to compare the light sensitivity of photocurrent for these two heterostructures, responsivity (*R*), *R* = [*J*_sc_ – *J*_dark V,0_]/*I*_p_, where *I*_p_ is the power density of the incident light. The *R* observed in the epitaxial BFCO with ZnO is ∼0.01 A/W, higher than the one obtained for epitaxial BFCO with no ZnO (∼4.6 × 10^–3^ A/W; see Table 1). These *R* values are even higher than that extracted from reported epitaxial BFO with metal electrodes and no interface layer (1.5 × 10^–4^ A/W).^46,47^ It is clear that the use of both the ZnO interface layer and ITO electrodes improves responsivity. On the other hand, the estimated *R* from the analogous polycrystalline system (ITO/ZnO/poly-BFO/Pt),^27^ ∼0.01 A/W, is similar to the one reported in this work, but the fact that they attribute the large photoresponse to the electron–hole pair generated in ZnO hinders a direct comparison of the influence of BFO crystallinity. Finally, the *R* found in our ZnO/BFCO films is larger than those in other ZnO/ferroelectric perovskite oxide heterojunctions.^48^

Remarkably, the operational stability of the BFCO devices with no encapsulation at ambient conditions has been monitored for 2 months, displaying reproducible performances (Figure 3d). This confirms the high stability and robustness of the all-oxide BFCO devices and contrasts with those of the non-encapsulated hybrid perovskites.^49^

Now, we concentrate on the macroscopic ferroelectric properties of the heterostructure with and without ZnO, which show large remnant polarizations (*P*_r_) along the (001) pseudocubic direction of ∼68 μC/cm^2^, Figure S6, similar to that reported for epitaxial films and larger than the polycrystalline films.^50−53^ The *P*–*E* loops are strongly shifted toward the positive voltage direction and reveal the presence of an imprint electric field (*E*_imp_) of 160 kV/cm pointing toward the top electrode, which could be attributed to the asymmetric top–bottom contact configuration of our systems.^54^ Also, *P*–*E* and *J*–*E* hysteresis loop measurements have been performed using a symmetric top–top configuration, that is contacting only ITO electrodes, revealing no signatures of the imprint electric field (see Figure S7), as has been previously reported for ferroelectric BFCO-based and BaTiO_3_ films.^34,54^

**Figure 4:**
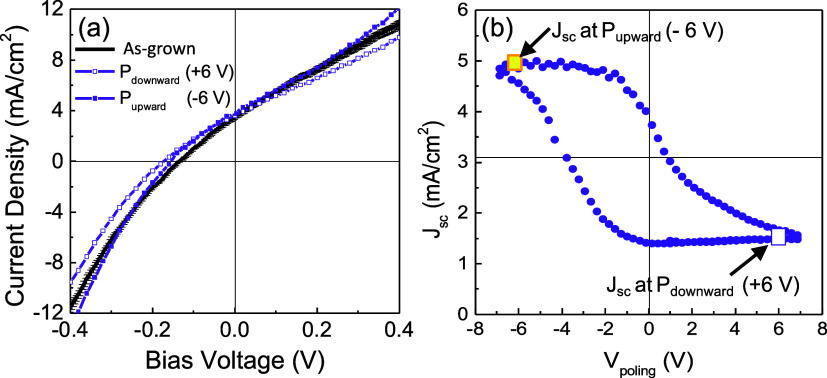
(a) *J*–*V* curve of the ITO/ZnO/BFCO/LSMO//STO heterostructure collected under illumination in as-grown, after downward (+6 V) and upward (−6 V) prepolarization with a time delay of ∼10 s. (b) Corresponding *J*_sc_ dependence with *V*_poling_ collected under illumination with a time delay of 0.1 s after the application of *V*_poling_ for 1 s.

Next, to investigate the role of the ferroelectric polarization on the photoresponse in a ZnO/BFCO-based device, two different polarization states have been induced to BFCO and compared to the as-grown state. According to the *E*_c_ found in the P–E loops, triangular prepoling voltages (*V*_poling_) of ±6 V were applied to the ITO top electrode for 0.2 s to induce the upward (−6 V) and downward (+6 V) polarization states. The polarization state is defined upward if the polarization direction points to the top electrode and downward if it points to the bottom electrode. The *J*–*V* curves were collected under illumination after 10 s of sample prepoling, i.e., 10 s delay time (detailed in Figure S8). From Figure 4a, it is first noted that the sign of the current density does not change after prepolarization, implying that the sense of the current flow is insensitive to the polarization direction.^13,55,56^

Nonetheless, a mild modulation is identified in the *J*–*V* by the polarization direction when compared with the as-grown with the prepoled systems. The variation observed in *J* in the upward polarization state compared to the downward polarization is consistent with the asymmetry in the Schottky barriers. Table 2 summarizes the *J*_sc_ and *V*_oc_ values for the three different states. This subtle modulation is attributed to the use of a long delay time after applying *V*_poling_ (10 s), which favors the back-switching of the FE domains because of the presence of imprint fields.^57,58^ To minimize the back-switching effects, determination of *J*_sc_ at zero bias has been carried out at different *V*_poling_ up to ±6.5 V using a shorter delay time of 0.1 s, as shown in Figure 4b. Details of the measurement protocol are depicted in Figure S8.^9,57^ Under these measurement conditions, a much larger modulation of *J*_sc_ is identified between the two prepolarized states, 68%, reaching a *J*_sc_ of ∼5.0 mA/cm^2^ and an IPCE of 6.3%.

**Table 2:** Comparison of *J*_sc_ and *V*_oc_ from *J*–*V* Characteristics of the ITO/ZnO/BFCO/LSMO/STO System in As-Grown, Upward, and Downward Polarization States (*V*_poling_ ± 6 V) with 10 s Delay Time

delay time (s)	polarization state	*J*_sc_ ± 0.1 (mA/cm^2^)	*V*_oc_ ± 0.01 (V)
	as-grown	3.5	–0.14
10	upward (−6 V)	3.8	–0.15
10	downward (+6 V)	3.6	–0.17

Returning to Figure 3a, the changes observed in the photoresponse between BFCO and BFCO/ZnO are further discussed based on the extracted energy band diagrams by means of XPS measurements.^42^

First, the “bulk-like” layers, ITO, ZnO, BFCO, and LSMO, have been investigated by measuring the valence band maxima (VBM) and the binding energy of the characteristic core levels (In 3d, Sn 3d, Zn 2p, Bi 4f, and Sr 3d) relative to the VBM^9^ (see sample and experiment details in the Experimental Section and Figures S9 and S10). Note that the extracted VBM values and binding energies are in agreement with those reported in the literature for the films with the same composition and prepared using the same deposition techniques.^44,59−64^

Then, different interfaces were prepared and investigated, namely, BFCO (5 nm)/LSMO, ITO (3 nm)/BFCO, ZnO (5 nm)/BFCO, and (3 nm ITO)/ZnO. Following a similar methodology, XPS spectra of the selected core levels together with the valence band were probed and compared to the core level binding energies of the bulk-like layer (Figure S9). It was observed that the intensities of the core levels for the buried layers dropped compared to the bare bulk-like layers, but it still allowed the identification of binding energy positions. In order to construct the band diagram and, in particular, the conduction band minimum, the energy gaps (*E*_g_) of BFCO (2.6 eV), ZnO (3.4 eV), and ITO (2.8 eV) have been used.^44,65^

**Figure 5:**
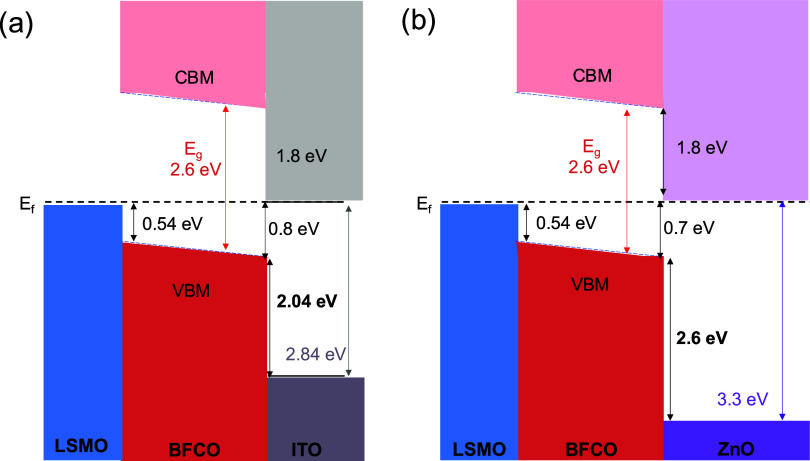
Schematic representation of the resulting energy band structure extracted from the XPS referred to as the Fermi level. Heterojunction of (a) LSMO/BFCO/ITO and (b) LSMO/BFCO/ZnO. Relevant VBM, valence band offset, and *E*_g_ are indicated.

A schematic representation of the energy band structure focused on the ITO/BFCO and ZnO/BFCO interfaces is depicted in Figure 5. It is noted that the *E*_F_–*E*_VBM_ values for the BFCO/ITO and BFCO/ZnO interfaces are 0.8 and 0.7 eV, respectively. Previous determination of reduction potentials and defect energy levels in BFCO films revealed a Fermi level position of *E*_F_–*E*_VBM_ = 1.1 eV from the clean BFCO surface.^59^ This Fermi level position was shifted to 0.45 eV upon oxygen plasma exposure and attributed to a change in the oxidation state of cobalt from II to III. Then, after water exposure, the *E*_F_–*E*_VBM_ could be further shifted to 0.8 eV, and it was limited by the partial reduction of cobalt to Co^2+^, the origin of the Fermi level pinning.^59,66^ Based on this observation, it is hypothesized that the Co in our BFCO layer is mainly in the 3+ oxidation state, having low Fermi energy (low oxygen vacancy concentration). Interface formation with ZnO or ITO will lead to downward band bending in BFCO toward the interface, eventually resulting in the reduction of Co^3+^ to Co^2+^ when a Fermi level of 0.8 eV above the VBM is reached. Consequently, the reduction of Co can be avoided in the case of ZnO (*E*_F_ is 0.7 eV above VBM) but not in the case of ITO (0.8 eV above VBM). Unfortunately, the intensity of the Co 2p level is too low after deposition of ZnO and ITO to determine the oxidation state of Co directly from the spectra. As the reduced Co^2+^ will act as a recombination center at the interface, the different Fermi level positions are consistent with the higher photocurrent density for the cell with the ZnO interlayer. An analogous situation is actually encountered in the case of chalcogenide Cu(In,Ga)(S,Se)_2_ thin film solar cells upon modifying the interfaces with buffer layers.^26^ It was found that the incorporation of buffer layers suppressed the formation of defects, the source of Fermi level pinning, and therefore showed that less band bending was more favorable for the working cell.^67^

From the ZnO/ITO interface, the formation of a barrier due to a slight upward band bending toward ITO (−0.4 eV) is identified (Figure S9 and Table S1). A similar behavior has been observed in other studies.^68,69^ The barrier will act as a contact injection barrier and may only influence the fill factor due to contact resistance.^70^ Thus, for the heterostructure with ZnO, the increase in *J*_sc_ will be mainly governed by the BFCO/ZnO interface.

Finally, also from this XPS experimental data, a built-in potential (*V*_BI,XPS_) of +0.16 eV emerges across ZnO/BFCO/LSMO, whereas a *V*_BI,XPS_ of +0.26 eV is calculated for ITO/BFCO/LSMO, both with the same sign (*V*_BI_ > 0). Based on these values, it would be expected that the current will flow from the top to the bottom electrode and should be *J*_sc_ > 0. In this line, the voltage required to cancel this photogenerated current, i.e., *V*_oc_, should be <0. These data agree well with the experimental results obtained from the *J*–*V* curves in Figure 3, in which *J*_sc_ > 0 and *V*_oc_ < 0. Importantly, the calculated value for *V*_BI,XPS_ (ITO/BFCO/LSMO) is faintly larger than that for V_BI,XPS_ (ZnO/BFCO/LSMO), being consistent with the trend observed for *V*_oc_ from *J*–*V* (see Table 1).

## Conclusions

In this work, the fabrication of an all-oxide device based on an epitaxial, photoferroelectric BFCO active layer is presented, and the role of the ZnO interface layer in ferroelectricity and photoresponse is investigated.

Importantly, the robustness of the device is demonstrated by monitoring reliable performance for up to 2 months. The measurements of the coupled ferroelectricity–photoresponse show that the sense of the current flow in ZnO/BFCO systems is insensitive to the polarization direction. Then, it is demonstrated that *J*_sc_ can be modulated up to ∼68% between two prepolarized states (+6 and −6 V), displaying an IPCE as high as 6.3% under monochromatic light (405 nm). Our epitaxial BFCO/ZnO system shows increased responsivity compared to the analogous system without ZnO and to other ferroelectric perovskite oxide heterojunctions. The extracted energy band diagrams from XPS provide useful insights into the photoresponse *J*–*V* measurements. The presence of the ZnO layer lowers the Fermi level for BFCO/ZnO and can suppress recombination, resulting in an increase in *J*_sc_. This behavior is tentatively attributed to the presence of less Co^2+^ species compared to the system with no ZnO. Exploring band alignment with compatible compositions that suppress defect formation and interface recombination will provide further guidance toward the design and use of photoferroelectric systems for niche optoelectronic applications.

## Supporting Information Available

Optical transmittance measurements and transmittance map simulation by the transfer matrix method; additional *J*–*V* analysis on ITO/ZnO/BFCO/LSMO//STO with different ZnO thicknesses; X-ray diffraction analysis of the oxide heterostructures with a 2D detector; *P*–*E*, *J*–*V*, and *J*–*V*_poling_ of ITO/ZnO/BFCO/LSMO//STO; *J*–*V* of ITO/ZnO/LSMO//STO; and details on the XPS core level emission lines and band alignment study (PDF)
