# Supplementary material for: Interface Engineering in All-Oxide Photovoltaic Devices Based on Photoferroelectric BiFe0.9Co0.1O3 Thin Films
Source: ACS Appl Electron Mater. 2024 Nov 13;6(11):8251–9. doi: 10.1021/acsaelm.4c01533 (PMC11603610; doi:10.1021/acsaelm.4c01533)
Supplement: Supplementary file 1 — el4c01533_si_001.pdf [file el4c01533_si_001.pdf]

Supporting Information for Interface engineering in all-oxide photovoltaic devices based on photoferroelectric $BiFe_{0.9}Co_{0.1}O_3$ thin films

Pamela Machado,[†] Pol Salles,[†] Alexander Frebel,[†,‡] Gabriele De Luca,[†,¶] Eloi Ros,[§] Christian Hagendorf,[‖] Ignasi Fina,[†] Joaquim Puigdollers,[§] and Mariona Coll[*,†]

†*Institut de Ciència de Materials de Barcelona ICMAB-CSIC, Campus UAB, Bellaterra 08193, Spain*

‡*Department of Materials and Geo Sciences, Technische Universität Darmstadt, Surface Science Division, Jovanka-Bontschits-Straße 2, D-64287 Darmstadt, Germany*

¶*Catalan Institute of Nanoscience and Nanotechnology (ICN2), Campus UAB, 08193, Bellaterra, Barcelona, Spain*

§*Departament d'Enginyeria Electrònica, Universitat Politècnica de Catalunya, Jordi Girona 1-3, Barcelona, 08034, Spain*

‖*Fraunhofer Center for Silicon-Photovoltaics CSP, Otto-Eissfeldt-Strasse 12, Halle (Saale) 06120, Germany*

E-mail: mcoll@icmab.es

Thickness study

The thickness of the different components of the heterostructure was verified to be 100 nm BFCO on 10 nm LSMO by TEM cross-section analysis acquired from FEI Titan STEM (high resolution) at Halle, Figure S1(a). Then, the thicknesses of ZnO and ITO were extracted from control samples being placed in the reaction chamber at the same time as the heterostructure. It was obtained 20 nm for ZnO on STO single crystal by XRR and 70 nm for ITO on glass by profilometry, S1(b) and (c), respectively.

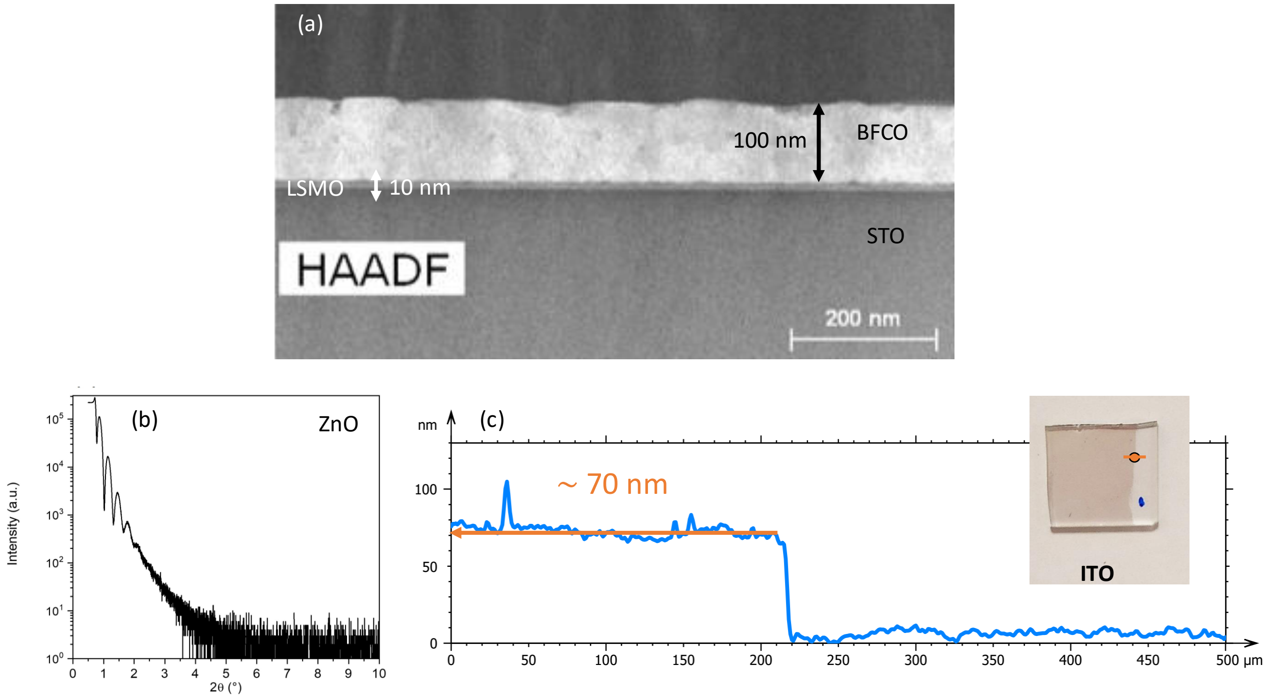

Figure S1: (a) Cross- section HAADF image from 100 nm BFCO/10 nm LSMO//STO film and (b)XRR of 20 nm ALD-ZnO deposited on $SrTiO_3$ substrate. (c) height profile extracted from profilometry of sputtered ITO on glass.

Optical properties

The optical properties of the oxide architectures have been investigated by UV-Vis spectroscopy transmittance (%T) curves measured in the wavelength range of 200 to 800 nm, Figure S2(a). From the transmittance spectra of bare STO substrate (grey curve) it can

be observed a strong absorption for photon energies above 3.2 eV, consistent with its band gap (see inset).[1,2] The %T drops in LSMO//STO (blue curve) in congruence with LSMO thickness and oxygen processing conditions previously reported.[3] The addition of BFCO film (brown curve) exhibits no changes in the maximum transmittance, however a red-shift is revealed in the absorption edge, as it can be observed in the absorbance spectra (inset), being in reasonable agreement with the band gap reported for high quality epitaxial BFCO films.[2,4] With the incorporation of ITO (red curve) and the selective layer ITO/ZnO (purple), the absorption edge slightly shifts to the violet and blue regions and higher transmittance is shown, features that are characteristic of these coatings.[5–8] The inset shows the absorbance spectra measured in the same conditions. Note that the use of one-side polished STO substrates attenuate the signal in a factor of ∼2.7 between 400 and 800 nm resulting in low %T value, see Figure S2(b). In addition, the thickness of the ITO and ZnO layers have been previously optimized to obtain maximum transmittance and compared to the average transmittance map simulation using the transfer matrix method, Figure S2(c).[9] From this simulation it can be observed that the maximum of transmittance should be obtained for systems with 50-70 nm of ITO and ∼ 10-20 nm ZnO coatings. Importantly, the increase in T% of ∼15% with the integration of these ITO and ZnO coatings is in well agreement with that observed in Figure S2(a).

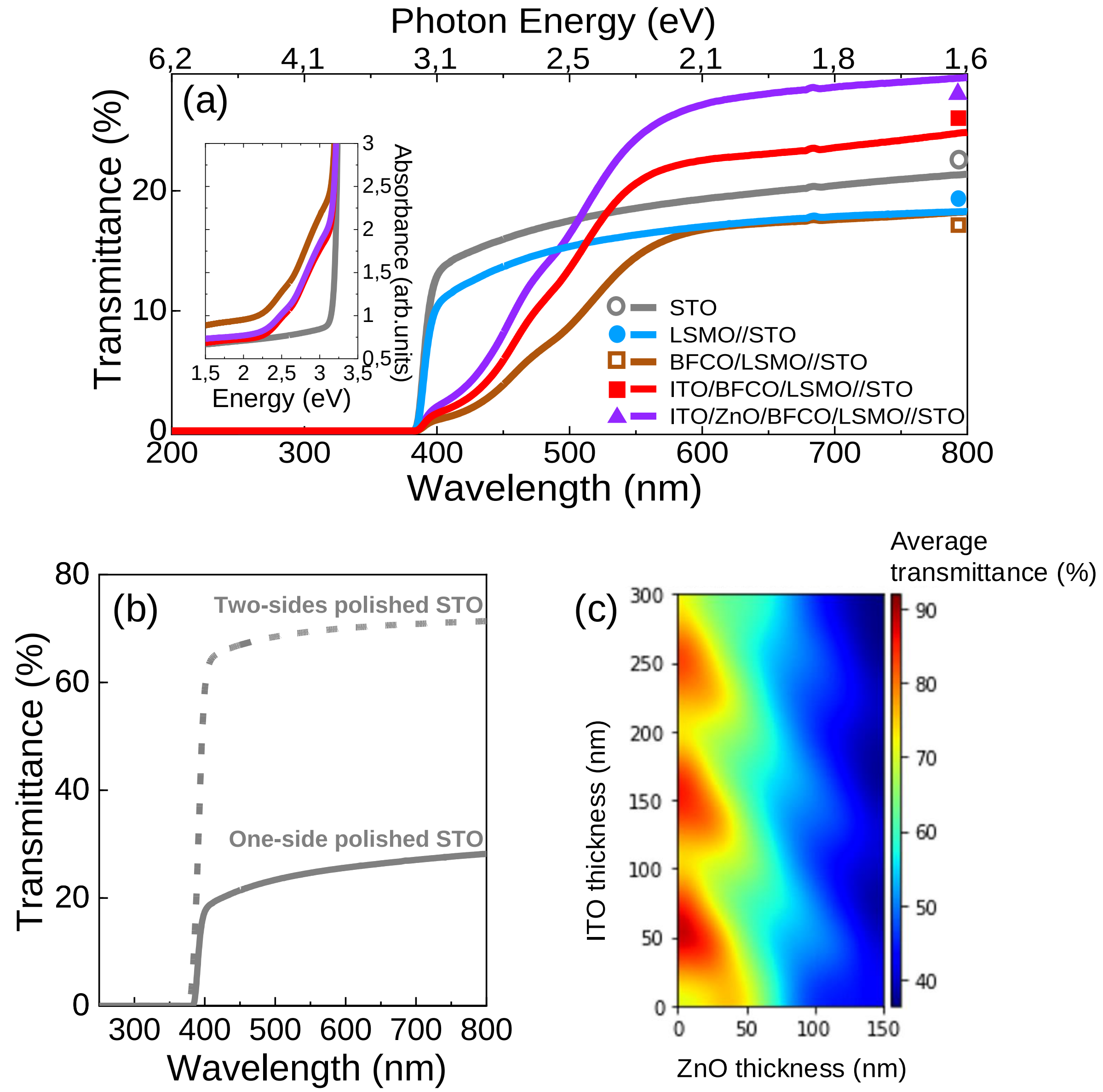

Figure S2: Optical characterization of BFCO-based systems. (a) Optical transmittance spectra of the oxide heterostructures BFCO/LSMO//STO (brown), ITO/BFCO/LSMO//STO (red), ITO/ZnO/BFCO/LSMO//STO (purple) and the bottom electrode LSMO//STO (blue) and STO substrate (grey). The inset shows the absorbance spectra of the systems. (b) Comparative optical transmittance spectra of one-side polished (001)-STO (solid line) and two-sides polished (001)-STO substrate (dashed line). (c) Transmittance map simulation of ITO/ZnO/BFCO system in function of ITO and ZnO thickness at 400 nm irradiance and obtained from transfer matrix model.

Optimization ZnO thickness

The J-V characteristics of the ITO/ZnO/BFCO/LSMO//STO system with different ZnO thicknesses between 4 - 130 nm have been measured under illumination with a monochromatic laser of 405 nm and irradiance of 350 mW/cm^2, revealing optimized photoresponse with improved J_{sc} and V_{oc} values for 20 nm ZnO, see Figure S3.

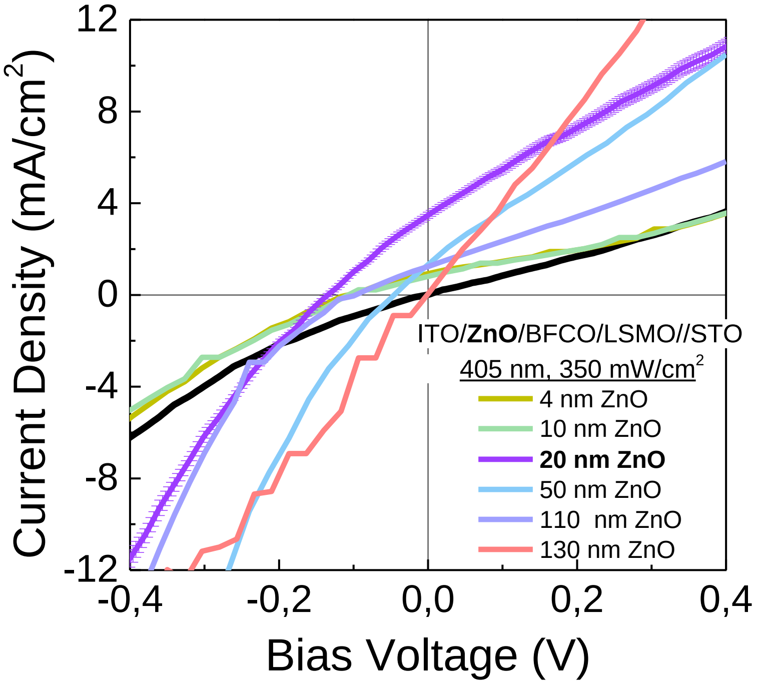

Figure S3: Comparative J-V characteristics of ITO/ZnO/BFCO/LSMO//STO system with ZnO thickness of 4 nm, 10 nm, 20 nm, 50 nm, 110 nm and 130 nm, recorded under illumination with a monochromatic laser of 405 nm and irradiance of 350 mW/cm^2.

Heterostructure Crystallinity

The crystallinity and phase purity of BFCO thin film on different oxide architectures has been studied by means of X-Ray Diffraction (XRD) using Bruker D8 Advance (GADDS) diffractometer equipped with a 2D detector. Figure S4(a) shows the θ-2θ 2D-XRD pattern for ITO/BFCO/LSMO//STO system; the two bright spots at 22.5° and 46.5° correspond to (00l) Bragg reflections of STO substrate. No Bragg reflection is observed for 10 nm

LSMO thin film due to its small thickness. The two peaks at 22.5° and 45° observed in the integrated 2θ scan correspond to (001) and (002) reflections of BFCO, confirming its epitaxial growth on (00l)-LSMO//STO. An additional weak spot, marked with a square symbol, is observed at 32° which corresponds to typical bismuth and iron oxide rich phases as Bi_2O_3 or Bi_2FeO_9.[4,10] ITO is not observed because of its amorphous nature arising from the room temperate and low power density deposition conditions.[11] Figure S4(b) displays the θ-2θ 2D-XRD patterns for ITO/20 nm ZnO/BFCO/LSMO//STO heterostructure where the typical Bragg reflections for ZnO are not identified because of the small film thickness. In order to depict the polycrystalline nature of ZnO in these heterostructures, the θ-2θ 2D-XRD patterns for ITO/130 nm ZnO/BFCO/LSMO//STO heterostructure is shown in panel (c) where three rings at 2θ= 31.9°, 34.3° and 36.4° are clearly identified and correspond to ZnO.[12]

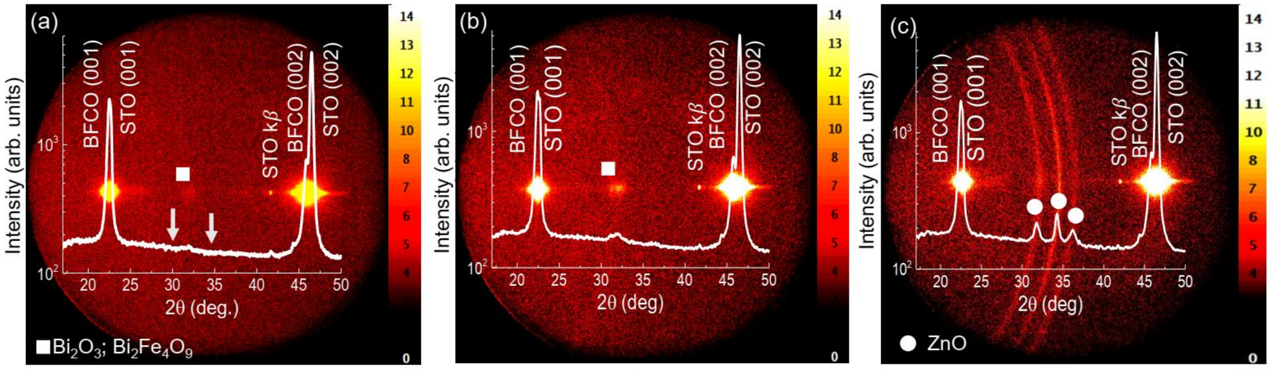

Figure S4: 2D-XRD frames overlapped with the corresponding integrated intensity 2θ scan of (a) ITO/BFCO/LSMO//STO and (b) ITO/20 nm ZnO/BFCO/LSMO//STO and (c)ITO/130 nm ZnO/BFCO/LSMO//STO oxide architectures.

J-V and P-E characterization

Photoresponse measurements for the ITO/ZnO/LSMO//STO system have been carried out. Figure S5 presents the J-V characteristic curves with linear dependence that passes through J-V axes origin in both dark and under illumination conditions, indicating that no photo-generation occurs in ZnO.

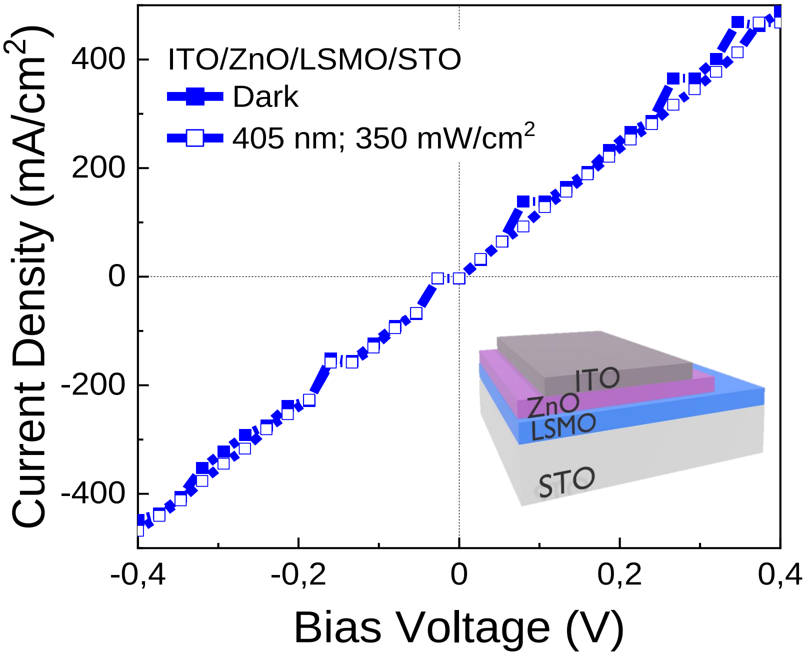

Figure S5: J-V characteristic curves of ITO/ZnO/LSMO//STO recorded in dark and under illumination with a monochromatic laser of 405 nm and irradiance of 350 mW/cm^2 from the top of ITO electrode.

Figure S6 shows the FE polarization-electric field (P-E) and the current density-electric field (J-E) hysteresis loops recorded at 2 kHz in dark and room temperature conditions. The presence of current switching peaks in J-E confirms the FE character and the nearly rectangular shape of the P-E loops indicates low leakage through the heterostructure.[13–15] In the two heterostructures here studied, the negative and positive coercive electric field (E_c) are ∼ -370 kV/cm and ∼530 kV/cm, respectively,

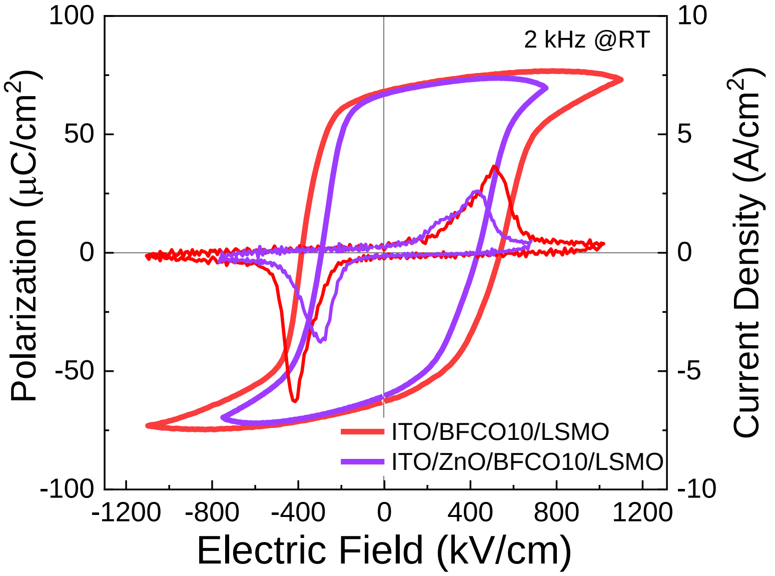

Figure S6: (a) J-E and P-E hysteresis loops recorded at 2 kHz and room temperature for the two studied systems.

Figure S7 shows P-E and J-E hysteresis loops of ITO/ZnO/BFCO/LSMO//STO in top-top configuration, that is contacting only ITO electrodes. These loops have been recorded at lower frequency of 500 Hz, room temperature and in dark conditions. A symmetric P-E loop with $E_c \sim 385$ mV/cm in both positive and negative directions is observed, demonstrating no signatures of imprint electric field, probably attained to the symmetric configuration of ITO-ITO electrodes that could allow for the formation of identical Schottky barriers at the ITO/ZnO/BFCO interfaces, as it has been previously reported for $BaTiO_3$ ferroelectric oxide.[16]

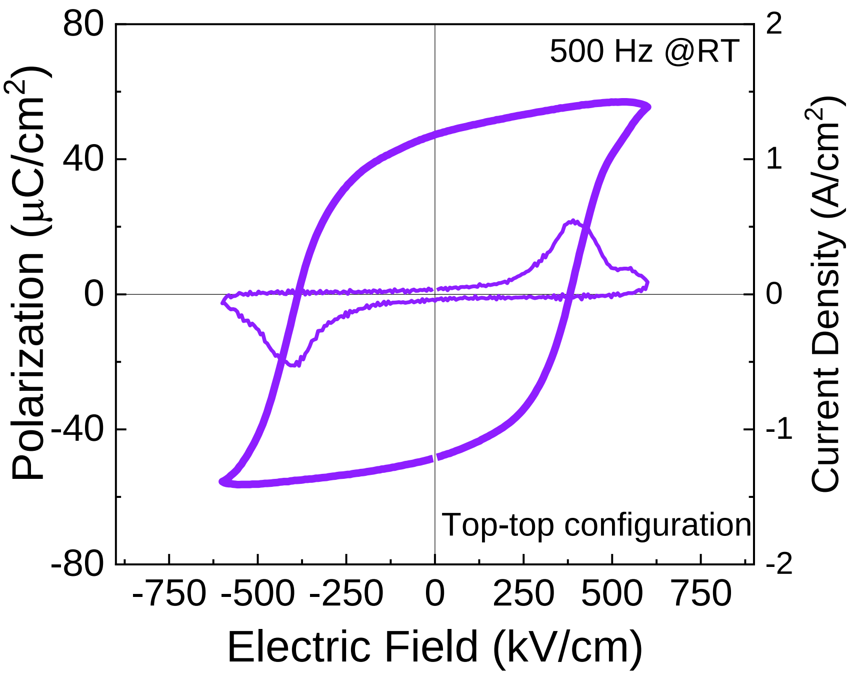

Figure S7: J-E and P-E hysteresis loops recorded at 500 Hz, room temperature and dark conditions for ITO/ZnO/BFCO/LSMO//STO system in top-top configuration.

Figure S8 shows the two measurement protocol followed to investigate the influence of ferroelectricity on photoresponse. First, a triangular and unipolar V_{poling} of up to $\pm$ 6 V was applied to the top of ITO electrode for 0.2 s and, after $\sim$ 10 s (time delay), a voltage sweep of $\pm$ 0.4 V was applied under illumination to collect the J-V curve, Figure S8 (a). On the other hand, a train of unipolar V_{poling} of up to $\pm$ 6.5 V was applied to the top of ITO electrode for $\sim$ 1 s and the J_{sc} was measured under illumination with a time delay of 0.1 s to record the J_{sc}-V_{poling} dependence, Figure S8(b).

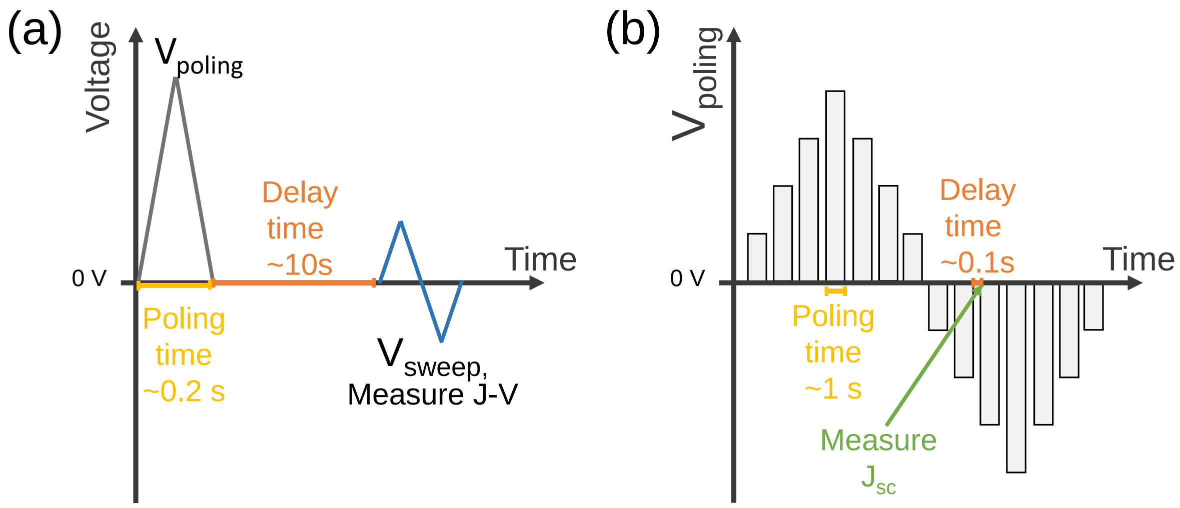

Figure S8: Protocol to measure J_{sc} of ITO/ZnO/BFCO/LSMO//STO heterostructure with (a) 10 s delay time after the application of V_{poling} on top of ITO and (b) 0.1 s delay time after the application of V_{poling} for 1 s on top of ITO. The illustrations are not scaled and do not show the V_{poling} values.

Study of the band energy alignment

Figure S9 shows the XPS core level emission lines and valence band of the investigated surfaces and interfaces. Starting from the analysis of the single layers, the core level binding energy for Bi $4f_{7/2}$ is at 158.61 eV which matches Bi^{3+} in BFO.[17] The core level of Sr $3d_{5/2}$ is at 132.37 eV, typical for Sr^{2+} in LSMO.[18] Next, both In $3d$ and Sn $3d$ have been recorded for ITO as an overlapping between Bi $4d$ and In $3d$, and Sn $3d$ with Zn LMM Auger was anticipated for the interface systems and would hinder the direct comparison of the core level binding energies. Sn $3d_{3/2}$ appeared at 486.73 eV and In $3d_{3/2}$ at 444.83 eV, in agreement with Sn^{4+} and In^{3+} in ITO, respectively.[19] Finally, Zn $2p_{3/2}$ is centered at 1022.33 eV matching Zn^{2+} in ZnO.[20] The values of the binding energies for the core levels and valence band maxima (VBM) referred to the Fermi energy (E_F), see Figure S10, are listed in Table S1.

Then, the interfaces of the system ITO/BFCO/LSMO and ITO/ZnO/BFCO/LSMO are

evaluated, Figure S9. Upon the formation of the interface, the intensity of the emission lines of the buried layers is reduced but allows to identify the binding energies which are listed in Table S1. We consider LSMO as a metallic electrode and therefore it has no band bending. The difference in binding energy positions between the single layer and interface system (ΔE_{CL}=($E_{CL,interface} - E_{CL,singlelayer}$)) is then used to determine the shift of the VBM and CBM which are also included in Table S1. The energy band diagrams for the ITO/BFCO/LSMO and ITO/ZnO/BFCO/LSMO systems have been proposed in **Figure 5** of the main manuscript, where Fermi Level, energy gap (E_g) and barrier heights are indicated. Details on the calculation of the ΔE_{VBM}, conduction band minima and offset (CBM, ΔE_{CBM}) and built in potential (V_{BI})[21,22] can be found in Equations S1-S3.

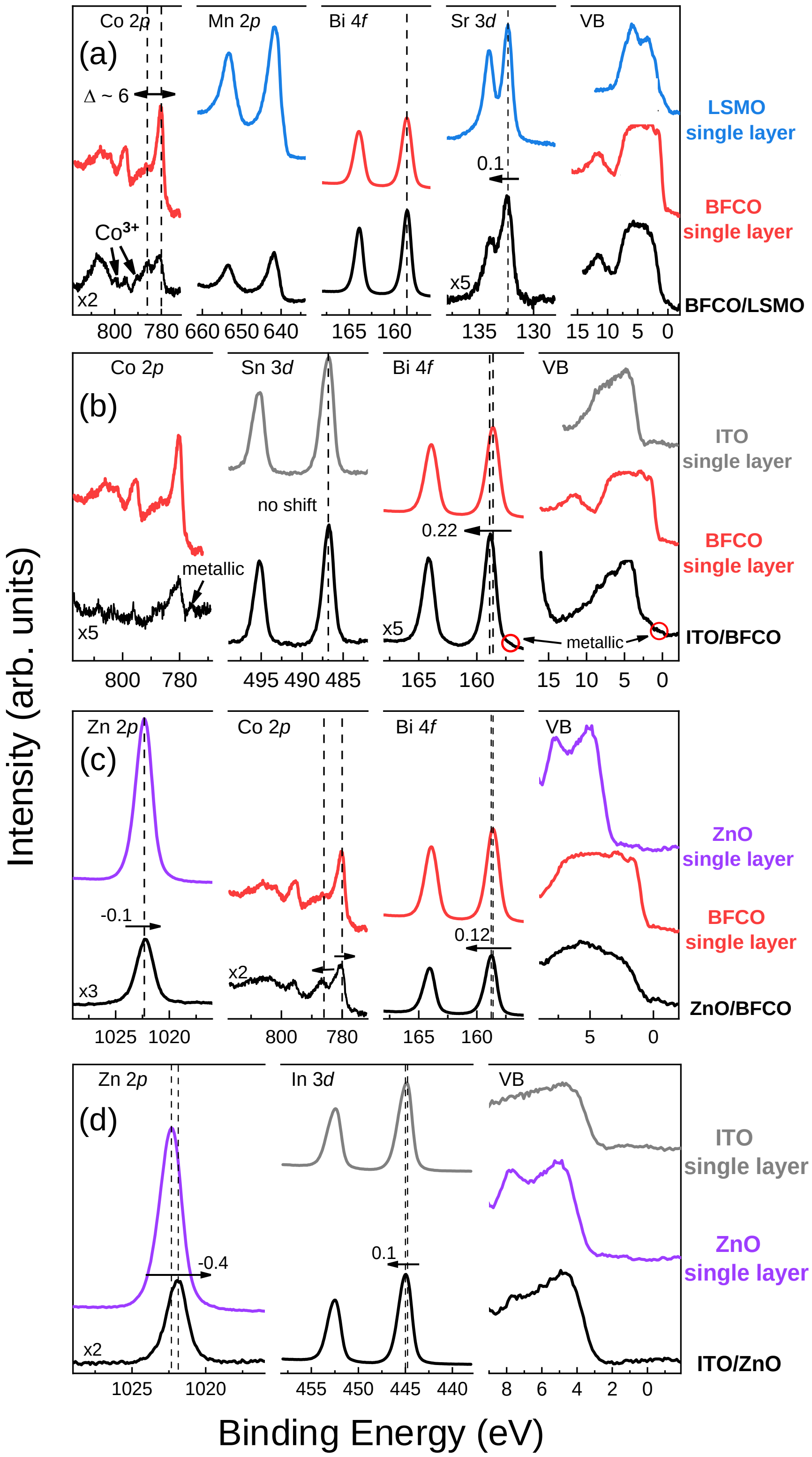

Figure S9: The XPS core level emission lines and valence band (VB) of the investigated surfaces and interfaces. (a) LSMO, BFCO single layers and BFCO/LSMO interface. (b) ITO, BFCO single layers, and ITO/BFCO interface. (c) ZnO, BFCO single layers, and ZnO/BFCO interface. (d) ZnO, ITO single layers and ITO/ZnO interface.

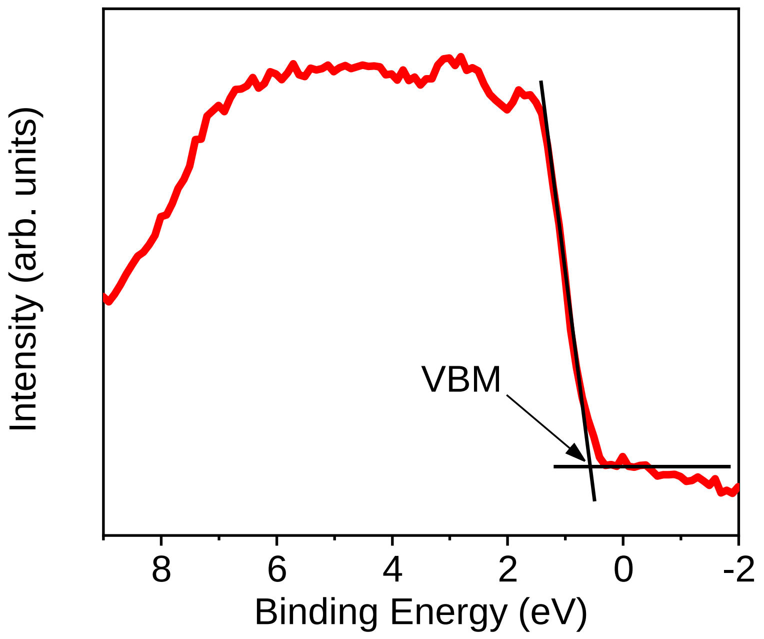

Figure S10: VBM determination for the 100 nm BFCO/10 nm LSMO//STO system.

Here below are described the equations to extract the ΔE_{VBM}, ΔE_{CBM} and V_{BI}.

Equation S1

$\Delta E_{VBM} = (E_{CL,single\text{-}layer1} - E_{VBM1}) - (E_{CL,single\text{-}layer2} - E_{VBM2}) - (E_{CL,layer1,interface} - E_{CL,layer2,interface})$

where E_{CL} is the core level binding energy and 1 and 2 refer to upper and inferior layers, respectively.

Equation S2

$\Delta E_{CBM} = (E_{CL,single\text{-}layer1} - E_{CBM1}) - (E_{CL,single\text{-}layer2} - E_{CBM2}) - (E_{CL,layer1,interface} - E_{CL,layer2,interface})$

$(E_{CBM} = E_{VBM} - E_g)$

Equation S3

$V_{BI,XPS} = E_{CL,interface2} - E_{CL,interface1}$

For example,

$V_{BI,XPS}(LSMO/BFCO/ITO) = E_{Bi4f(BFCO/ITO)} - E_{Bi4f(BFCO/LSMO)} = +0.26$ eV

$V_{BI,XPS}(LSMO/BFCO/ZnO) = E_{Bi4f(BFCO/ZnO)} - E_{Bi4f(BFCO/LSMO)} = +0.16$ eV

Table S1: Values extracted for the binding energies for specific core levels, VBM, CBM.

(a) BFCO, LSMO single layer and BFCO/LSMO interface

(eV)	Co $2p_{3/2}$/Sat.	Mn $2p_{3/2}$	Bi $4f_{7/2}$	Sr $3d_{5/2}$	VBM	CBM
BFCO	779.9 / 786		158.61		0.58	-2.0
LSMO		641.42		132.37	-0.34	
BFCO/LSMO	~780/ ~786	641.72	158.57	132.47	0.54/-0.24	-2.1/
$\Delta_{CL}(E_{CL,interface} - E_{CL,single\ layer})$			-0.04	0.10		

(b) BFCO, ZnO single layer and ZnO/BFCO interface

(eV)	Zn $2p_{3/2}$	Co $2p_{3/2}$/Sat.	Bi $4f_{7/2}$	VBM	CBM
BFCO		779.9/ 786	158.61	0.58	-2.0
ZnO	1022.33			3.40	0
ZnO/BFCO	1022.23	780.12 / 787.77	158.73	3.3/0.70	-0.1/-1.9
$\Delta_{CL}(E_{CL,interface} - E_{CL,single\ layer})$	-0.10		0.12		

(c) BFCO, ITO single layer and ITO/BFCO interface

(eV)	Co $2p_{3/2}$/Sat.	Sn $3d_{5/2}$	Bi $4f_{7/2}$	VBM	CBM
BFCO	779.9 / 786		158.61	0.58	-2.0
ITO		486.73		2.84	0
ITO/BFCO	~780/ ~786	486.73	158.83	2.84/0.80	0/-1.8
$\Delta_{CL}(E_{CL,interface} - E_{CL,single\ layer})$		0.00	0.22		

(d) ZnO, ITO single layer and ITO/ZnO interface

[eV]	Zn $2p_{3/2}$	In $3d_{5/2}$	VBM	CBM
ZnO//Si	1022.33		3.40	0
ITO//Si		444.83	2.84	0
ITO/ZnO	1021.93	444.93	2.94/3.10	0.1/-0.4
$\Delta_{CL}(E_{CL,interface} - E_{CL,single\ layer})$	-0.40	0.10		

References

(1) Cardona, M. Optical Properties and Band Structure of $SrTiO_3$ and $BaTiO_3$. *Phys. Rev.* **1965**, *140*, A651–A655.

(2) Sando, D.; Carrétéro, C.; Grisolia, M. N.; Barthélémy, A.; Nagarajan, V.; Bibes, M. Revisiting the Optical Band Gap in Epitaxial $BiFeO_3$ Thin Films. *Advanced Optical Materials* **2018**, *6*, 1700836.

(3) Martino, M.; Cesaria, M.; Caricato, A. P.; Maruccio, G.; Cola, A.; Farella, I. La07Sr03MnO3 thin films deposited by pulsed laser ablation for spintronic applications. *Physica Status Solidi A, Applications and Materials Science* **2011**, *208*, 1817–1820.

(4) Machado, P.; Scigaj, M.; Gazquez, J.; Rueda, E.; Sánchez-Díaz, A.; Fina, I.; Gibert-Roca, M.; Puig, T.; Obradors, X.; Campoy-Quiles, M.; Coll, M. Band Gap Tuning of Solution-Processed Ferroelectric Perovskite $BiFe_{1\breve{}x}Co_xO_3$ Thin Films. *Chemistry of Materials* **2019**, *31*, 947–954, PMID: 30828131.

(5) Feng, H.-J.; Yang, K.; Deng, W.; Li, M.; Wang, M.; Duan, B.; Liu, F.; Tian, J.; Guo, X. The origin of enhanced optical absorption of the BiFeO3/ZnO heterojunction in the visible and terahertz regions. *Phys. Chem. Chem. Phys.* **2015**, *17*, 26930–26936.

(6) Amalathas, A. P.; Alkaisi, M. M. Effects of film thickness and sputtering power on properties of ITO thin films deposited by RF magnetron sputtering without oxygen. *Journal of Materials Science: Materials in Electronics* **2016**, *27*, 11064–11071.

(7) Li, K.; Zhang, W.; Guo, K.; Cui, R.; Deng, C.; Wang, X. Modulating light absorption and multiferroic properties of BiFeOsub3/sub-based ferroelectric films by the introduction of ZnO layer. *Materials Research Express* **2022**, *9*, 036402.

(8) Abdyldayeva, N.; Beisenkhanov, N. ZnO-ITO multi-layered structure on Si substrate with prospective usage as antireflective covering for solar cells. *Materials Today: Proceedings* **2022**, *49*, 2516–2520, The 8th International Conference on Nanomaterials and Advanced Energy Storage Systems (INESS-2020).

(9) Katsidis, C. C.; Siapkas, D. I. General transfer-matrix method for optical multilayer systems with coherent, partially coherent, and incoherent interference. *Appl. Opt.* **2002**, *41*, 3978–3987.

(10) Machado, P.; Caño, I.; Menéndez, C.; Cazorla, C.; Tan, H.; Fina, I.; Campoy-Quiles, M.; Escudero, C.; Tallarida, M.; Coll, M. Enhancement of phase stability and optoelectronic

performance of $BiFeO_3$ thin films via cation co-substitution. *J. Mater. Chem. C* **2021**, *9*, 330–339.

(11) Huang, M.; Hameiri, Z.; Aberle, A. G.; Mueller, T. Comparative study of amorphous indium tin oxide prepared by pulsed-DC and unbalanced RF magnetron sputtering at low power and low temperature conditions for heterojunction silicon wafer solar cell applications. *Vacuum* **2015**, *119*, 68–76.

(12) Guziewicz, E.; Godlewski, M.; Krajewski, T.; Wachnicki, ; Szczepanik, A.; Kopalko, K.; Wójcik-Głodowska, A.; Przeździecka, E.; Paszkowicz, W.; Łusakowska, E.; Kruszewski, P.; Huby, N.; Tallarida, G.; Ferrari, S. ZnO grown by atomic layer deposition: A material for transparent electronics and organic heterojunctions. *Journal of Applied Physics* **2009**, *105*, 122413.

(13) Lee, D.; Baek, S. H.; Kim, T. H.; Yoon, J.-G.; Folkman, C. M.; Eom, C. B.; Noh, T. W. Polarity control of carrier injection at ferroelectric/metal interfaces for electrically switchable diode and photovoltaic effects. *Phys. Rev. B* **2011**, *84*, 125305.

(14) Wang, L.; Jin, K.-j.; Ge, C.; Wang, C.; Guo, H.-z.; Lu, H.-b.; Yang, G.-z. Electro-photo double modulation on the resistive switching behavior and switchable photoelectric effect in $BiFeO_3$ films. *Applied Physics Letters* **2013**, *102*, 252907.

(15) Fang, L.; You, L.; Zhou, Y.; Ren, P.; Shiuh Lim, Z.; Wang, J. Switchable photovoltaic response from polarization modulated interfaces in BiFeO3 thin films. *Applied Physics Letters* **2014**, *104*, 142903.

(16) Sharma, S.; Kumar, M.; Laref, A.; Siqueiros, J.; Herrera, O. R. Unravelling and controlling hidden imprint fields in ferroelectric capacitors. *Scientific Reports* **2016**, *6*, 25028(1:7).

(17) Bein, N. S.; Machado, P.; Coll, M.; Chen, F.; Makarovic, M.; Rojac, T.; Klein, A. Electrochemical Reduction of Undoped and Cobalt-Doped $BiFeO_3$ Induced by Water

Exposure: Quantitative Determination of Reduction Potentials and Defect Energy Levels Using Photoelectron Spectroscopy. *The Journal of Physical Chemistry Letters* **2019**, *10*, 7071–7076, PMID: 31664832.

(18) Horiba, K.; Chikamatsu, A.; Kumigashira, H.; Oshima, M.; Wadati, H.; Fujimori, A.; Lippmaa, M.; Kawasaki, M.; Koinuma, H. Temperature-dependence of the electronic structure of $La_{1-x}Sr_xMnO_3$ thin films studied by in situ photoemission spectroscopy. *Journal of Electron Spectroscopy and Related Phenomena* **2007**, *156-158*, 375–378.

(19) Gassenbauer, Y.; Schafranek, R.; Klein, A.; Zafeiratos, S.; Hävecker, M.; Knop-Gericke, A.; Schlögl, R. Surface states, surface potentials, and segregation at surfaces of tin-doped In_2O_3. *Physical Review B* **2006**, *73*.

(20) Säuberlich, F.; Fritsche, J.; Hunger, R.; Klein, A. Properties of sputtered ZnO films and its interfaces with CdS. *Thin Solid Films* **2003**, *431-432*, 378–381.

(21) Waldrop, J. R.; Grant, R. W.; Kowalczyk, S. P.; Kraut, E. A. Measurement of semiconductor heterojunction band discontinuities by x–ray photoemission spectroscopy. *Journal of Vacuum Science & Technology A: Vacuum, Surfaces, and Films* **1985**, *3*, 835–841.

(22) Sheng, Y.; Mirjolet, M.; Villa, M.; Gàzquez, J.; Santiso, J.; Klein, A.; Fraxedas, J.; Fontcuberta, J. Band Alignment and Photoresponse of $LaFeO_3$-Based Heterojunctions. *Phys. Rev. Appl.* **2023**, *19*, 024001.
